# Comparing Pathological Risk Factors for Dementia between Cognitively Normal Japanese and Americans

**DOI:** 10.3390/brainsci11091180

**Published:** 2021-09-08

**Authors:** Chendi Cui, Aya Higashiyama, Brian J. Lopresti, Masafumi Ihara, Howard J. Aizenstein, Makoto Watanabe, Yuefang Chang, Chikage Kakuta, Zheming Yu, Chester A. Mathis, Yoshihiro Kokubo, Tetsuya Fukuda, Victor L. Villemagne, William E. Klunk, Oscar L. Lopez, Lewis H. Kuller, Yoshihiro Miyamoto, Akira Sekikawa

**Affiliations:** 1 Department of Epidemiology, Graduate School of Public Health, University of Pittsburgh, Pittsburgh, PA 15261, USA; chc217@pitt.edu (C.C.); kullerl@edc.pitt.edu (L.H.K.); 2 Department of Preventive Cardiology, National Cerebral and Cardiovascular Center, Suita 564-8565, Japan; ahigashi@ncvc.go.jp (A.H.); makotow@ncvc.go.jp (M.W.); ykokubo@ncvc.go.jp (Y.K.); miyamoty@ncvc.go.jp (Y.M.); 3 Department of Hygiene, Wakayama Medical University, Wakayama 641-0011, Japan; 4 Department of Radiology, University of Pittsburgh, Pittsburgh, PA 15213, USA; brianl@pitt.edu (B.J.L.); zhy39@pitt.edu (Z.Y.); mathca@upmc.edu (C.A.M.); 5 Department of Neurology, National Cerebral and Cardiovascular Center, Suita 564-8565, Japan; ihara@ncvc.go.jp (M.I.); chikage.kakuta@ncvc.go.jp (C.K.); 6 Department of Psychiatry, University of Pittsburgh, Pittsburgh, PA 15213, USA; aizensteinhj@upmc.edu (H.J.A.); victor.villemagne@pitt.edu (V.L.V.); wek1@pitt.edu (W.E.K.); 7 Department of Neurological Surgery, University of Pittsburgh, Pittsburgh, PA 15213, USA; yuc2@pitt.edu; 8 Department of Radiology, National Cerebral and Cardiovascular Center, Suita 564-8565, Japan; tetsuyaf@ncvc.go.jp; 9 Department of Neurology, University of Pittsburgh, Pittsburgh, PA 15213, USA; lopezol@upmc.edu; 10 Open Innovation Center, National Cerebral and Cardiovascular Center, Suita 564-8565, Japan

**Keywords:** neuroimaging, biomarker, Japanese, amyloid PET imaging, magnetic resonance imaging

## Abstract

The Alzheimer’s Disease Neuroimaging Initiative showed that Japanese had significantly lower brain Aβ burden than Americans among a cognitively normal population. This cross-sectional study aimed to compare vascular disease burden, Aβ burden, and neurodegeneration between cognitively normal elderly Japanese and Americans. Japanese and American participants were matched for age (±4-year-old), sex, and Apolipoprotein E (*APOE*) genotype. Brain vascular disease burden and brain Aβ burden were measured using white matter lesions (WMLs) and ^11^C-labeled Pittsburgh Compound B (PiB) retention, respectively. Neurodegeneration was measured using hippocampal volumes and cortical thickness. A total of 95 Japanese and 95 Americans were recruited (50.5% men, mean age = 82). Compared to Americans, Japanese participants had larger WMLs, and a similar global Aβ standardized uptake value ratio (SUVR), cortical thickness and hippocampal volumes. Japanese had significantly lower regional Aβ SUVR in the anterior ventral striatum, posterior cingulate cortex, and precuneus. Cognitively normal elderly Japanese and Americans had different profiles regarding vascular disease and Aβ burden. This suggests that multiple risk factors are likely to be involved in the development of dementia. Additionally, Japanese might have a lower risk of dementia due to lower Aβ burden than Americans. Longitudinal follow-up of these cohorts is warranted to ascertain the predictive accuracy of these findings.

## 1. Introduction

Vascular disease burden, amyloid-β (Aβ) burden, and neurodegeneration are major pathological risk factors of dementia [1,2]. The relative prevalence of these major risk factors might differ across populations despite a similar prevalence of dementia across developed countries [3]. Japanese have a notably high vascular disease burden. Prevalence of hypertension is higher in Japan than in other developed countries [4,5]. Japanese have had higher stroke mortality and higher prevalence of silent brain infarcts than Americans [6,7]. In addition, the Alzheimer’s Disease Neuroimaging Initiative (ADNI) reported a lower brain Aβ burden among Japanese than among Americans [8].

The primary aim of this study was to compare brain vascular disease burden, Aβ burden, and neurodegeneration between Japanese and American elderly with normal cognition. Therefore, we measured white matter lesions (WMLs), Aβ deposition, cortical thickness, and hippocampal volume among Japanese and American elderly who were cognitively normal and matched on age, sex, and apolipoprotein E (*APOE*) genotype. Our secondary aim was to compare the regional Aβ burden between Japanese and American elderly, as we previously reported that the regional Aβ burden is a more sensitive marker than the global Aβ burden, especially in the early stages of Aβ deposition [9]. We aimed to corroborate previous findings that Japanese had a higher vascular disease burden and lower global and regional Aβ burdens than Americans.

## 2. Materials and Methods

### 2.1. Study Population

**Figure 1:**
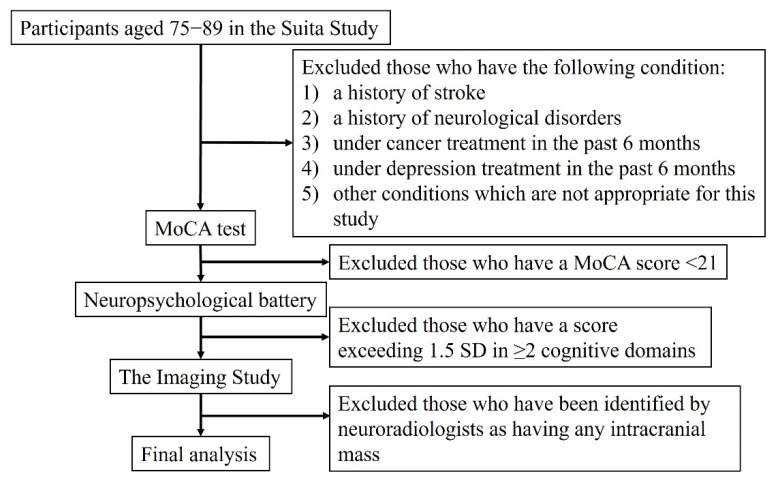
Inclusion and exclusion criteria of Japanese participants.

Between 2016 and 2019, a total of 95 participants who were cognitively normal and aged 75–89 were recruited from the Suita Study, a large population-based prospective cohort study in Japan [10] (Figure 1). This age group was selected because individuals with normal cognition in this age group are likely to have high vascular disease burden and Aβ burden, but have not yet developed the dementia phenotype [7,11]. A total of 210 participants aged 75–89 without any history of stroke, psychiatric or neurological disorders, any cancer or depression treatment in the past 6 months, or other conditions such as substance abuse, liver or renal disease, were invited to the study. Through a series of neuropsychological tests, 108 participants were identified as having normal cognition and invited to participate in the imaging study. A total of 95 participants were included in the final analysis, after excluding 13 participants (suboptimal imaging (*n* = 4), intracranial mass (*n* = 3), and lost to follow-up (*n* = 6)). For each Japanese participant, a matched American participant with normal cognition was identified from a cohort at the University of Pittsburgh PET research center. Japanese and American participants were matched for age (±4-year-old), sex, and *APOE* genotype. One exception was an 80-year-old Japanese participant with *APOE* ε2/ε4 who was matched to an 87-year-old American participant with *APOE* ε2/ε4 due to the rareness of this genotype. This study was approved by the Institutional Review Boards of the University of Pittsburgh and the National Cerebral and Cardiovascular Center (NCVC) in Japan. Informed consent was obtained from all of the participants in this study.

### 2.2. Normal Cognition

The neuropsychological batteries used to identify cognitively normal individuals in the American cohort have been previously reported [12]. The neuropsychological batteries used in the Japanese cohort were comparable to those used in the American cohort (Figure 1). The Montreal Cognitive Assessment (MoCA) was initially used for screening. Participants with a score <21 were excluded from further examinations. In the second step, a detail neuropsychological battery was administered to characterize normal cognition. The neuropsychological battery consisted of the Wechsler Adult Intelligence Scale-III (WAIS-III) digit span, WAIS-III block design, WAIS-III digit symbol, trail making tests A and B, word fluency category (animals and vegetables), word fluency letter (start with “ka”), Alzheimer’s Disease Assessment Scale-Cognitive subscale (ADAS-Cog) word list (immediate and delayed), Rey complex figure test (immediate, recall, copy), Raven’s colored progressive matrices, Boston naming test, and Stroop test. The result of each test in the battery was classified as normal or abnormal (1.5 standard deviation (SD) below the mean value among individuals with comparable age and education), based on normative data [12,13,14]. Normal cognition was defined as abnormal results in ≤1 cognitive domain. The Mini-Mental State Examination (MMSE) score for the Japanese participants was calculated from the MoCA score, according to Roalf et al. [15].

### 2.3. Other Variables

Education level was ascertained using questionnaires. Height and body weight were measured in the fasting state. Body mass index (BMI) was calculated as weight (kg) divided by the square of the height (m^2^). Polymorphisms of the *APOE* gene were determined by the GTS-7000 system (Shimadzu, Kyoto, Japan) at the NCVC [16]. This system detects single-nucleotide polymorphisms on direct polymerase chain reaction amplification with no requirement for DNA extraction. *APOE* ε4 carriers were defined as those with *ε*2/*ε*4, *ε*3/*ε*4, or *ε*4/*ε*4 genotypes, and non-carriers were defined as those with *ε*2/*ε*2, *ε*2/*ε*3, or *ε*3/*ε*3 genotypes.

### 2.4. Magnetic Resonance Imaging (MRI)

Participants were scanned on a 3-Tesla Siemens MAGNETOM Trio scanner. A structural T1-weighted magnetization-prepared rapid gradient echo (MPRAGE, TR/TE = 2300/2.98 ms, TI = 900 ms, 1 mm × 1 mm × 1.2 mm sagittal acquisition) sequence was used for PET-MR image registration, brain segmentation and parcellation for PET image sampling. For assessing white matter hyperintensities, we used a T2-weighted fluid-attenuated inversion recovery (FLAIR-T2) sequence (TR/TE = 9002/56 ms Ef, TI = 2200 ms, NEX = 1) with an interleaved acquisition (48 slices, 3 mm, no gap). To obtain a good signal-to-noise ratio, an average of 4 acquisitions was used. A fuzzy-connectedness algorithm was used to segment the WML from each individual’s FLAIR-T2 images [17]. The volume of WML is presented as the proportion of the total brain volume. All of the acquired images were analyzed at the University of Pittsburgh.

After excluding those who had missing data, a total of 95 Japanese and 70 American participants were included in the comparison of WMLs. Abnormal WMLs were defined as >75th percentile of volume normalized to the total brain volume [1]. A total of 95 Japanese and 24 American participants were included in the comparison of hippocampal volumes. Hippocampal atrophy was defined as either the right or left hippocampus <25th percentile [1]. A total of 95 Japanese participants and 76 American participants who had 3T scans were included in the comparison of cortical thickness. The cortical thickness was determined by a FreeSurfer-based analysis shown to be highly reproducible [18]. The mean cortical thickness outcome measure was a composite of four individual cortical thickness ROIs previously shown to be highly related to Alzheimer’s disease pathology: entorhinal cortex, inferior temporal cortex, middle temporal cortex, and fusiform gyrus [19]. Abnormal cortical thickness was defined as <2.74 mm [19].

### 2.5. Positron-Emission Tomography Imaging

PET imaging was performed as previously described [20]. Briefly, participants were intravenously injected with 15mCi of [^11^C] Pittsburgh compound-B (PiB) over 20 s. A 20-min PET scan (4 × 5-min frames) was acquired, beginning 50 min after PiB injection [21]. All PET scans were acquired in 3D-mode using a Siemens Biograph mCT PET/CT scanner (22.1 cm axial field-of-view, reconstructed image resolution ~5 mm full-width-at-half-maximum (FWHM)) and reconstructed using filtered back-projection. Prior to radiotracer administration, a low-dose (<20 mrem) non-diagnostic CT scan (19 mA, 120 kVp, 1.0 mm pitch) was acquired for attenuation correction of PET emission data. All scans were acquired in three-dimensional mode and reconstructed using filtered back projection. Other standard PET data corrections required for quantitation (e.g., scatter, dead-time) were applied during the reconstruction process.

PET images were processed and analyzed using a semi-automated analysis pipeline based on FreeSurfer (v5.3) software as previously described [22,23]. Specific PiB retention was indexed by the standardized uptake value ratio (SUVR) using cerebellar grey matter as reference [24]. A global cortical index of total Aβ load was determined, based upon a weighted average of nine sub-regions (GBL9) relevant to Aβ pathology (anterior cingulate, posterior cingulate, insula, superior frontal cortex, orbitofrontal cortex, lateral temporal cortex, parietal, precuneus and ventral striatum). Global Aβ positivity was defined as GBL9 ≥ 1.346, determined using a sparse k-means clustering method applied to a sample of 62 cognitively normal controls [9]. The SUVR outcome measure indexing PiB retention has been shown to be highly reproducible (±~5% across regions) [24]. All of the PET images were analyzed at the University of Pittsburgh.

### 2.6. Statistical Analysis

Continuous participant characteristics were summarized as either mean ± SD or median (interquartile range) for normal and skewed distributions, respectively. Categorical characteristics were summarized as a percentage (n). Basic demographic characteristics were compared between Japanese and American participants. In addition, global PiB standardized uptake value ratio (SUVR), WMLs, cortical thickness, and hippocampal volume were compared. Further, we compared the prevalence of dichotomized biomarkers (i.e., WMLs > 75th percentile, hippocampal atrophy, abnormal cortical thickness, and PiB positivity) between Japanese and Americans. In addition, the regional PiB SUVRs were compared between Japanese and Americans. Lastly, global and regional PiB SUVRs were compared among *APOE* ε4 non-carriers between the Japanese and Americans. *t*-test and Wilcoxon rank sum tests were used for variables with normal distribution, and skewed distribution, respectively, and a Chi-square test was used for categorical variables. All analyses were performed using SAS version 9.4 (Cary, NC, USA), with statistical significance set at *p* < 0.05.

## 3. Results

As per the inclusion criteria, Japanese and American participants were comparable regarding age, sex, *APOE* genotype, and global cognition (Table 1). Compared to the Americans, the Japanese participants had a lower BMI (22.4 ± 3.0 vs. 26.5 ± 4.4, *p*-value < 0.01) and were less educated (12.6 ± 2.3 vs. 14.4 ± 2.6, *p*-value < 0.01, in Japanese and Americans, respectively).

The comparison of pathological risk factors for dementia is shown in Table 2. Japanese had significantly larger WMLs than Americans (*p*-value < 0.01). There were no significant differences in global Aβ burden. The global PiB SUVR was 1.16 and 1.19 for Japanese and Americans, respectively (*p*-value = 0.42). There were no significant differences in cortical thickness (*p*-value = 0.11) or hippocampal volumes (*p*-value = 0.62) between Japanese and Americans.

There were 30.0% (*n* = 27) Japanese and 17.1% (*n* = 12) Americans with high WMLs (*p*-value = 0.06). The prevalence of PiB positivity was 29.5% (*n* = 28) and 34.7% (*n* = 33) among Japanese and Americans, respectively (*p*-value = 0.44). The prevalence of abnormal cortical thickness (*p*-value = 0.47) and hippocampal atrophy (*p*-value = 0.90) was similar between Japanese and Americans.

The regional Aβ burden among Japanese and American participants is presented in Table 3. Compared to Americans, Japanese had a significantly lower Aβ burden in the anterior ventral striatum (median (interquartile range, IQR): Japanese = 1.09 (1.02, 1.30), Americans = 1.20 (1.10, 1.84), *p*-value < 0.01), posterior cingulate cortex (median (IQR): Japanese = 1.29 (1.20, 1.63), Americans = 1.35 (1.25, 1.79), *p*-value = 0.02), and precuneus (median (IQR): Japanese = 1.21 (1.14, 1.65), Americans = 1.32 (1.17, 1.91), *p*-value < 0.01). The Aβ burden in other brain regions was similar between Japanese and Americans. Further analysis of *APOE* ε4 non-carriers showed similar results (Table 4).

## 4. Discussion

The present study compared three important pathological risk factors for dementia among cognitively normal elderly in Japan and in the US, who were matched for age, sex, and *APOE* genotype. Japanese had larger WMLs than Americans. Japanese and Americans had similar global Aβ, cortical thickness and hippocampal volumes. Japanese had a significantly lower regional Aβ burden than Americans in the anterior ventral striatum, posterior cingulate cortex, and precuneus.

We have previously reported that WMLs, Aβ burden, and hippocampal volume were each independently associated with incidence of mild cognitive impairment and dementia [1,2]. Those with more than one of the three risk factors had a significantly higher risk of mild cognitive impairment and dementia than those with one or none of these risk factors [1,2]. We did not observe a significant association among WMLs, Aβ burden, and hippocampal volume in our study population, which was similar to results of previous studies [25,26,27]. However, cortical thickness was negatively associated with Aβ burden and WMLs. These associations were similar to what has been previously reported [28,29]. The present study found that two populations, with normal cognition and matched for age, sex, and *APOE* genotype, could have different risk factor profiles. Our results show that different cohorts could have different pathological risk profiles, for example, the Japanese population had higher WML pathological burden, but lower Aβ burden. These different profiles between Japanese and Americans directly demonstrate that WMLs and Aβ burden both contribute independently to cognitive function in the elderly. Further, this supports the concept of multiple underlying pathologies leading to the phenotypical manifestation of dementia.

This study showed that Japanese participants had larger WMLs than Americans. This finding is consistent with the fact that hypertension is more prevalent in Japan than in other developed countries, [4,5], which is largely due to the much higher dietary intake of salt in Japan [30]. Our finding is also consistent with observations that Japanese have higher stroke mortality and prevalence of silent brain infarcts than Americans [6,7]. It is reported that Japanese have higher levels of atherosclerosis in the brain than Americans [31], despite a substantially lower burden of coronary artery atherosclerosis [32,33].

The ADNI study reported a lower prevalence of Aβ positivity in elderly Japanese with normal cognition compared to their American counterparts (23% vs. 44%, *p*-value < 0.001), despite Japanese supposedly being at higher risk of increased Aβ accumulation due to hypertension impairing Aβ clearance [8,34]. Even though the Japanese J-ADNI participants were 5 years younger than the American ADNI participants (age: 67.9 vs. 73.1, respectively; *p*-value < 0.001), the estimated difference in Aβ positivity prevalence is 7.1% between cognitively normal individuals aged 75 and 70 [11], which is much smaller than the observed difference of 21%. The present study also found a lower regional Aβ burden in Japanese elderly adults with normal cognition compared to their American counterparts.

This study is the first to compare the regional Aβ deposition between Japanese and Americans. In the two cohorts, which were matched for age, sex, and *APOE* genotype, the present study showed that Japanese, despite a similar global Aβ burden, had lower regional Aβ burdens than Americans in the anterior ventral striatum, posterior cingulate cortex, and precuneus. As we previously reported, regional measures are more sensitive than global measures [9]. The posterior cingulate cortex and precuneus are important regions of the default mode network [35] and are brain areas of early Aβ deposition [35]. Early striatal Aβ deposition has been associated with overproduction of Aβ [36]. Given the high vascular disease burden, Japanese are likely to have a higher risk of impaired Aβ clearance [37]. Therefore, lower regional Aβ burden in the anterior ventral striatum, posterior cingulate cortex, and precuneus might reflect lower Aβ production in Japanese participants.

The Japanese have the highest life expectancy among all developed countries [38] and a unique lifestyle, especially in terms of diet, with a high intake of marine omega-3 fatty acids [39], soy isoflavones [40] and salt [30]. Marine omega-3 fatty acids and soy isoflavones are nutrients which may be considered to be beneficial to brain health. A cross-sectional study reported that serum levels of docosahexaenoic acid (DHA), a major omega-3 fatty acid, were significantly inversely associated with Aβ deposition [41]. The inverse association between DHA and Aβ may be attributed to the fact that DHA suppresses Aβ production [42] and promotes Aβ clearance [43]. Regarding soy isoflavones, we and others reported that supplementation of soy isoflavones improved cognitive function, especially in the memory domains, based on evidence from the systematic review and meta-analysis of randomized controlled trials (RCTs) [44,45]. In addition, our systematic review and meta-analysis of RCTs found that supplementation of soy isoflavones reduced arterial stiffness [46]. It is noted that we have previously reported significant positive associations of arterial stiffness with Aβ deposition, disease progression [37,47] and dementia risk [48]. High salt intake was associated with an increased risk of hypertension and stroke [49]. Since the Japanese have a higher prevalence of hypertension and stroke, but similar prevalence of dementia, compared to Americans, Japanese would have lower prevalence of dementia than Americans if their salt intake was similar to that of Americans. Although the difference might be attributed to genetic differences between Japanese and Americans, the Japanese and American cohorts were matched according to *APOE* genotype, which is the major genetic determinant of dementia [50]. Accordingly, high intake of marine omega-3 fatty acids and soy isoflavones might be associated with a reduced risk of Aβ deposition and dementia.

The strengths of the study include the fact that we compared multiple important pathological risk factors for dementia in two different populations. The matching approach reduced the influence of confounders. In addition, the PET images and MRI scans of Japanese and American participants were read and analyzed at the same center. This study was not without limitations. Cognitively normal participants were identified retrospectively in the US cohort, although protocols to define normal cognition were comparable in both cohorts. Compared to Americans, Japanese participants were less educated and had a lower BMI. It is unclear if education and BMI have a direct impact over the pathological risk factors assessed here. Although the Japanese had lower levels of regional Aβ deposition, it remains unknown whether regional Aβ deposition would eventually progress to global Aβ positivity [51]. In addition, we have missing MRI data, especially for the American participants. Further, Japanese participants were from a population-based cohort, whereas American participants were recruited from clinical trials conducted by an academic institution. Given that participants of clinical trial-based studies are generally healthier than those of population-based studies [52,53], the difference in Aβ burden between Japanese and Americans might be greater than the observed difference in the present study. Finally, the cross-sectional nature of the study does not allow the relative prognostic accuracy of these pathological risk factors to be established.

## 5. Conclusions

The present study showed that, compared to Americans, Japanese elderly with normal cognition had larger WMLs, lower regional brain Aβ deposition, and similar levels of cortical thickness and hippocampal volume. Through observing different profiles of pathological risk factors between Japanese and American elderly with comparable cognitive function, this study demonstrated the importance of multiple factors: brain vascular disease burden, Aβ burden, and neurodegeneration, each of which likely independently and/or synergistically contributes to the risk of progressing to dementia. The potential difference in Aβ burden between Japanese and Americans warrants further investigation. A comparison of tau pathology between Japanese and Americans should be considered in future research. A follow-up study of this cohort assessing imaging and cognitive changes would provide valuable information on the progression of these biomarkers and whether the incidence of mild cognitive impairment and dementia is different between Japanese and Americans.

## Figures and Tables

**Table 1:** Characteristics of Japanese and American participants.

	Japanese (*n* = 95)	Americans (*n* = 95) ^a^	*p*-Value
Age in years, mean (SD)	81.7 (3.1)	82.2 (3.8)	0.30
BMI, kg/m^2^, mean (SD)	22.4 (3.0)	26.5 (4.4)	<0.01
Men, n (%)	48 (50.5)	48 (50.5)	1.00
Years of education, mean (SD)	12.6 (2.3)	14.4 (2.6)	<0.01
MMSE score, median (IQR) ^b^	29 (28, 29)	28 (27, 30)	0.40
*APOE*, n (%)			1.00
ε2/ε3	9 (9.5)	9 (9.5)	
ε2/ε4	1 (1.1)	1 (1.1)	
ε3/ε3	79 (83.2)	79 (83.2)	
ε3/ε4	6 (6.3)	6 (6.3)	

Abbreviations: *APOE*: *Apolipoprotein E* genotype; BMI: body mass index; IQR: interquartile range; MMSE: The Mini-Mental State Examination. Mean (SD) and *t*-test were used for age and years of education, which are normally distributed. Median (interquartile range) and Wilcoxon rank sum test were used for MMSE due to skewed distribution. ^a^: Japanese and Americans were matched on age, sex, and *Apolipoprotein E allele* status. ^b^: Skewed distribution. MMSE score for Japanese was calculated from MoCA score according to Roalf et al. [15].

**Table 2:** Pathological risk factors for dementia for Japanese and Americans.

	Japanese (*n* = 95)	Americans (*n* = 95) ^a^	*p*-Value
Continuous biomarker			
WMLs, median (IQR) ^b^	0.011 (0.006, 0.016)	0.005 (0.002, 0.011)	<0.01
Global PiB SUVR, median (IQR) ^b^	1.16 (1.11, 1.41)	1.19 (1.11, 1.64)	0.42
Cortical thickness, mm, mean (SD)	2.84 (0.15)	2.79 (0.18)	0.11
Hippocampal volume, mm^3^, mean (SD)	6680.03 (813.28)	6586.31 (838.61)	0.62
Dichotomized biomarker ^c^			
High WMLs, n (%)	27 (30.0)	12 (17.1)	0.06
PiB positivity, n (%)	28 (29.5)	33 (34.7)	0.44
Abnormal cortical thickness, n (%)	24 (25.3)	23 (30.3)	0.47
Hippocampal atrophy, n (%)	33 (34.7)	8 (33.3)	0.90

Abbreviations: PiB: Pittsburgh Compound B; IQR: interquartile range; WMLs: white matter lesions; SUVR: the standardized update value ratio. Mean (SD) and *t*-test were used for cortical thickness and hippocampal volume which are normally distributed. For WMLs, and global PiB SUVR, median (interquartile range) and Wilcoxon rank sum test were used due to skewed distribution. ^a^: Japanese and Americans were matched on age, sex, and *Apolipoprotein E allele* status. Among Americans, there were 70, 76, and 24 participants with WMLs, cortical thickness, and hippocampal data, respectively. ^b^: The distribution is skewed. ^c^: PiB positivity was defined as a global SUVR ≥1.346. Abnormal WMLs were defined as >75th percentile of volumes normalized to the total brain volume [1]. Abnormal cortical thickness was defined as <2.74 mm [19]. Hippocampal atrophy was defined as either the right or left hippocampus <25th percentile [1].

**Table 3:** Comparison of regional Aβ burden between Japanese and Americans.

	Japanese (*n* = 95)	Americans (*n* = 95) ^a^	*p*-Value ^b^
Regional PiB SUVR, median (IQR)			
Anterior Ventral Striatum	1.09 (1.02, 1.30)	1.20 (1.10, 1.84)	<0.01
Posterior Cingulate	1.29 (1.20, 1.63)	1.35 (1.25, 1.79)	0.02
Precuneus	1.21 (1.14, 1.65)	1.32 (1.17, 1.91)	<0.01
Anterior Cingulate	1.22 (1.14, 1.50)	1.27 (1.15, 1.85)	0.07
Insula	1.17 (1.11, 1.34)	1.17 (1.10, 1.58)	0.83
Lateral Temporal	1.15 (1.09, 1.36)	1.16 (1.07, 1.56)	0.64
Orbitofrontal	1.25 (1.17, 1.52)	1.23 (1.11, 1.72)	0.21
Parietal	1.14 (1.09, 1.40)	1.21 (1.08, 1.66)	0.37
Superior Frontal	1.14 (1.08, 1.46)	1.18 (1.09, 1.62)	0.24

Abbreviations: IQR: interquartile range; PiB: Pittsburgh Compound B; SUVR: the standardized update value ratio. ^a^: Japanese and Americans are matched on age, sex, and *Apolipoprotein E allele* status. ^b^: *p*-values are based on Wilcoxon rank sum test.

**Table 4:** Comparison of global and regional Aβ burden between Japanese and American *APOE ε4* non-carriers.

	Japanese (*n* = 88)	Americans (*n* = 88) ^a^	*p*-Value ^b^
Global PiB SUVR	1.16 (1.11, 1.37)	1.19 (1.10, 1.61)	0.43
Regional PiB SUVR, median (IQR)			
Anterior Ventral Striatum	1.08 (1.01, 1.23)	1.19 (1.10, 1.64)	<0.01
Posterior Cingulate	1.28 (1.20, 1.47)	1.34 (1.24, 1.75)	0.02
Precuneus	1.19 (1.13, 1.49)	1.28 (1.17, 1.82)	<0.01
Anterior Cingulate	1.22 (1.14, 1.46)	1.27 (1.15, 1.78)	0.05
Insula	1.16 (1.11, 1.31)	1.17 (1.10, 1.49)	0.72
Lateral Temporal	1.14 (1.09, 1.29)	1.15 (1.07, 1.52)	0.72
Orbitofrontal	1.24 (1.17, 1.50)	1.21 (1.11, 1.67)	0.22
Parietal	1.13 (1.08, 1.34)	1.18 (1.08, 1.63)	0.41
Superior Frontal	1.14 (1.08, 1.41)	1.16 (1.09, 1.58)	0.21

Abbreviations: *APOE*: *Apolipoprotein E* genotype; IQR: interquartile range; PiB: Pittsburgh Compound B; SUVR: the standardized update value ratio. ^a^: Japanese and Americans are matched on age, sex, and *Apolipoprotein E allele* status. ^b^: *p*-values are based on Wilcoxon rank sum test.

## Data Availability

The datasets used and/or analyzed during the current study are available from the corresponding author on reasonable request.

## Abbreviations

Aβ	amyloid beta
ADAS-Cog	Alzheimer’s Disease Assessment Scale-Cognitive subscale
ADNI	Alzheimer’s Disease Neuroimaging Initiative
*APOE*	Apolipoprotein E
BMI	Body mass index
DHA	docosahexaenoic acid
FWHM	full-width-at-half-maximum
IQR	interquartile range
MMSE	Mini-Mental State Examination
MoCA	Montreal Cognitive Assessment
MRI	Magnetic resonance imaging
NCVC	National Cerebral and Cardiovascular Center
PET	Positron emission tomography
PiB	Pittsburgh Compound B
RCTs	randomized controlled trials
SD	standard deviation
SUVR	standardized uptake value ratio
WAIS-III	Wechsler Adult Intelligence Scale-III
WMLs	white matter lesions

## References

[1] Lopez O.L., Becker J.T., Chang Y., Klunk W.E., Mathis C., Price J., Aizenstein H.J., Snitz B., Cohen A.D., DeKosky S.T. (2018). Amyloid deposition and brain structure as long-term predictors of MCI, dementia, and mortality. Neurology.

[2] Lopez O.L., Klunk W.E., Mathis C., Coleman R.L., Price J., Becker J.T., Aizenstein H.J., Snitz B., Cohen A., Ikonomovic M. (2014). Amyloid, neurodegeneration, and small vessel disease as predictors of dementia in the oldest-old. Neurology.

[3] Dodge H.H., Buracchio T.J., Fisher G.G., Kiyohara Y., Meguro K., Tanizaki Y., Kaye J.A. (2012). Trends in the Prevalence of Dementia in Japan. Int. J. Alzheimers Dis..

[4] Forouzanfar M.H., Liu P., Roth G.A., Ng M., Biryukov S., Marczak L., Alexander L., Estep K., Abate K.H., Akinyemiju T.F. (2017). Global Burden of Hypertension and Systolic Blood Pressure of at Least 110 to 115 mm Hg, 1990–2015. JAMA.

[5] Danaei G., Finucane M.M., Lin J.K., Singh G.M., Paciorek C.J., Cowan M.J., Farzadfar F., Stevens G.A., Lim S.S., Riley L.M. (2011). National, regional, and global trends in systolic blood pressure since 1980: Systematic analysis of health examination surveys and epidemiological studies with 786 country-years and 5·4 million participants. Lancet.

[6] Benjamin E.J., Virani S.S., Callaway C.W., Chamberlain A.M., Chang A.R., Cheng S., Chiuve S.E., Cushman M., Delling F.N., Deo R. (2018). Heart Disease and Stroke Statistics—2018 Update: A Report From the American Heart Association. Circulation.

[7] Vermeer S.E., Longstreth W.T., Koudstaal P.J. (2007). Silent brain infarcts: A systematic review. Lancet Neurol..

[8] Iwatsubo T., Iwata A., Suzuki K., Ihara R., Arai H., Ishii K., Senda M., Ito K., Ikeuchi T., Kuwano R. (2018). Japanese and North American Alzheimer’s Disease Neuroimaging Initiative studies: Harmonization for international trials. Alzheimer’s Dement. J. Alzheimers Assoc..

[9] Cohen A.D., Mowrey W., Weissfeld L.A., Aizenstein H., McDade E., Mountz J.M., Nebes R.D., Saxton J.A., Snitz B., DeKosky S. (2013). Classification of amyloid-positivity in controls: Comparison of visual read and quantitative approaches. NeuroImage.

[10] Kokubo Y., Okamura T., Yoshimasa Y., Miyamoto Y., Kawanishi K., Kotani Y., Okayama A., Tomoike H. (2008). Impact of Metabolic Syndrome Components on the Incidence of Cardiovascular Disease in a General Urban Japanese Population: The Suita Study. Hypertens. Res. Off. J. Jpn. Soc. Hypertens..

[11] Jansen W.J., Ossenkoppele R., Knol D.L., Tijms B.M., Scheltens P., Verhey F.R., Visser P.J., Aalten P., Aarsland D., Alcolea D. (2015). Prevalence of cerebral amyloid pathology in persons without dementia: A meta-analysis. JAMA.

[12] DeKosky S.T., Fitzpatrick A., Ives D.G., Saxton J., Williamson J., Lopez O.L., Burke G., Fried L., Kuller L.H., Robbins J. (2006). The Ginkgo Evaluation of Memory (GEM) study: Design and baseline data of a randomized trial of Ginkgo biloba extract in prevention of dementia. Contemp. Clin. Trials.

[13] Dodge H.H., Kita Y., Takechi H., Hayakawa T., Ganguli M., Ueshima H. (2008). Healthy Cognitive Aging and Leisure Activities among the Oldest Old in Japan: Takashima Study. J. Gerontol. Ser. A Boil. Sci. Med. Sci..

[14] Lopez O.L., Jagust W.J., DeKosky S.T., Becker J.T., Fitzpatrick A., Dulberg C., Breitner J., Lyketsos C., Jones B., Kawas C. (2003). Prevalence and classification of mild cognitive impairment in the Cardiovascular Health Study Cognition Study: Part 1. Arch. Neurol..

[15] Roalf D.R., Moberg P.J., Xie S.X., Wolk D.A., Moelter S.T., Arnold S.E. (2013). Comparative accuracies of two common screening instruments for classification of Alzheimer’s disease, mild cognitive impairment, and healthy aging. Alzheimers Dement. J. Alzheimers Assoc..

[16] Ohno Y., Kitahara H., Fujii K., Kohno Y., Ariyoshi N., Nishi T., Fujimoto Y., Kobayashi Y. (2018). High residual platelet reactivity after switching from clopidogrel to low-dose prasugrel in Japanese patients with end-stage renal disease on hemodialysis. J. Cardiol..

[17] Wu M., Rosano C., Butters M., Whyte E., Nable M., Crooks R., Meltzer C.C., Reynolds C.F., Aizenstein H.J. (2006). A fully automated method for quantifying and localizing white matter hyperintensities on MR images. Psychiatry Res..

[18] Tustison N.J., Cook P.A., Klein A., Song G., Das S.R., Duda J.T., Kandel B.M., van Strien N., Stone J.R., Gee J.C. (2014). Large-scale evaluation of ANTs and FreeSurfer cortical thickness measurements. NeuroImage.

[19] Jack C.R., Wiste H.J., Weigand S.D., Knopman D.S., Mielke M.M., Vemuri P., Lowe V., Senjem M.L., Gunter J.L., Reyes D. (2015). Different definitions of neurodegeneration produce similar amyloid/neurodegeneration biomarker group findings. Brain J. Neurol..

[20] Mathis C.A., Kuller L.H., Klunk W.E., Snitz B.E., Price J.C., Weissfeld L.A., Rosario B.L., Bs B.J.L., Saxton J.A., Aizenstein H.J. (2013). In vivo assessment of amyloid-β deposition in nondemented very elderly subjects. Ann. Neurol..

[21] McNamee R.L., Yee S.-H., Price J.C., Klunk W.E., Rosario B., Weissfeld L., Ziolko S., Berginc M., Lopresti B., DeKosky S. (2009). Consideration of Optimal Time Window for Pittsburgh Compound B PET Summed Uptake Measurements. J. Nuclear Med. Off. Publ. Soc. Nucl. Med..

[22] Fischl B., Salat D.H., Busa E., Albert M., Dieterich M., Haselgrove C., van der Kouwe A., Killiany R., Kennedy D., Klaveness S. (2002). Whole Brain Segmentation: Automated Labeling of Neuroanatomical Structures in the Human Brain. Neuron.

[23] Snitz B.E., Tudorascu D.L., Yu Z., Campbell E., Lopresti B.J., Laymon C.M., Minhas D.S., Nadkarni N.K., Aizenstein H.J., Klunk W.E. (2020). Associations between NIH Toolbox Cognition Battery and in vivo brain amyloid and tau pathology in non-demented older adults. Alzheimer’s Dement. Diagn. Assess. Dis. Monit..

[24] Lopresti B.J., Klunk W.E., Mathis C.A., Hoge J.A., Ziolko S.K., Lu X., Meltzer C.C., Schimmel K., Tsopelas N.D., DeKosky S.T. (2005). Simplified quantification of Pittsburgh Compound B amyloid imaging PET studies: A comparative analysis. J. Nuclear Med. Off. Publ. Soc. Nucl. Med..

[25] Trzepacz P.T., Hochstetler H., Yu P., Castelluccio P., Witte M.M., Dell’Agnello G., Degenhardt E.K., Initiative F.T.A.D.N. (2015). Relationship of Hippocampal Volume to Amyloid Burden across Diagnostic Stages of Alzheimer’s Disease. Dement. Geriatr. Cogn. Disord..

[26] Roseborough A., Ramirez J., Black S.E., Edwards J.D. (2017). Associations between amyloid β and white matter hyperintensities: A systematic review. Alzheimers Dement. J. Alzheimers Assoc..

[27] Legdeur N., Visser P.J., Woodworth D.C., Muller M., Fletcher E., Maillard P., Scheltens P., DeCarli C., Kawas C.H., Corrada M. (2019). White Matter Hyperintensities and Hippocampal Atrophy in Relation to Cognition: The 90+ Study. J. Am. Geriatr. Soc..

[28] Dickie D.A., Gardner K., Wagener A., Wyss A., Arba F., Wardlaw J.M., Dawson J., Collaborators V.-P. (2020). Cortical thickness, white matter hyperintensities, and cognition after stroke. Int. J. Stroke Off. J. Int. Stroke Soc..

[29] Becker J.A., Hedden T., Ba J.C., Bs J.M., Rentz D.M., Putcha D., Fischl B., Greve D.N., Marshall G.A., Salloway S. (2010). Amyloid-β associated cortical thinning in clinically normal elderly. Ann. Neurol..

[30] Gabriel A.S., Ninomiya K., Uneyama H. (2018). The Role of the Japanese Traditional Diet in Healthy and Sustainable Dietary Patterns around the World. Nutrients.

[31] Resch J.A., Okabe N., Kimoto K. (1969). Stroke: U.S. and Japan. Cerebral atherosclerosis. Geriatrics.

[32] Sekikawa A., Ueshima H., Kadowaki T., El-Saed A., Okamura T., Takamiya T., Kashiwagi A., Edmundowicz D., Murata K., Sutton-Tyrrell K. (2007). Less Subclinical Atherosclerosis in Japanese Men in Japan than in White Men in the United States in the Post-World War II Birth Cohort. Am. J. Epidemiol..

[33] Sekikawa A., Miyamoto Y., Miura K., Nishimura K., Willcox B.J., Masaki K.H., Rodriguez B., Tracy R.P., Okamura T., Kuller L.H. (2015). Continuous decline in mortality from coronary heart disease in Japan despite a continuous and marked rise in total cholesterol: Japanese experience after the Seven Countries Study. Int. J. Epidemiol..

[34] Langbaum J.B., Chen K., Launer L.J., Fleisher A.S., Lee W., Liu X., Protas H.D., Reeder S.A., Bandy D., Yu M. (2012). Blood pressure is associated with higher brain amyloid burden and lower glucose metabolism in healthy late middle-age persons. Neurobiol. Aging.

[35] Palmqvist S., Scholl M., Strandberg O., Mattsson N., Stomrud E., Zetterberg H., Blennow K., Landau S., Jagust W., Hansson O. (2017). Earliest accumulation of beta-amyloid occurs within the default-mode network and concurrently affects brain connectivity. Nat. Commun..

[36] Cohen A.D., McDade E., Christian B., Price J., Mathis C., Klunk W., Handen B.L. (2018). Early striatal amyloid deposition distinguishes Down syndrome and autosomal dominant Alzheimer’s disease from late-onset amyloid deposition. Alzheimer’s Dement. J. Alzheimers Assoc..

[37] Hughes T.M., Kuller L.H., Barinas-Mitchell E.J., McDade E.M., Klunk W.E., Cohen A.D., Mathis C.A., Dekosky S.T., Price J.C., Lopez O.L. (2014). Arterial stiffness and beta-amyloid progression in nondemented elderly adults. JAMA Neurol..

[38] Ikeda N., Saito E., Kondo N., Inoue M., Ikeda S., Satoh T., Wada K., Stickley A., Katanoda K., Mizoue T. (2011). What has made the population of Japan healthy?. Lancet.

[39] Sekikawa A., Steingrimsdottir L., Ueshima H., Shin C., Curb J.D., Evans R.W., Hauksdottir A.M., Kadota A., Choo J., Masaki K. (2012). Serum levels of marine-derived n-3 fatty acids in Icelanders, Japanese, Koreans, and Americans—A descriptive epidemiologic study. Prostaglandins Leukot. Essent. Fat. Acids.

[40] Klein M.A., Nahin R.L., Messina M.J., Rader J.I., Thompson L.U., Badger T.M., Dwyer J.T., Kim Y.S., Pontzer C.H., Starke-Reed P.E. (2010). Guidance from an NIH Workshop on Designing, Implementing, and Reporting Clinical Studies of Soy Interventions. J. Nutr..

[41] Yassine H.N., Feng Q., Azizkhanian I., Rawat V., Castor K., Fonteh A.N., Harrington M., Zheng L., Reed B.R., DeCarli C. (2016). Association of Serum Docosahexaenoic Acid with Cerebral Amyloidosis. JAMA Neurol..

[42] Bazinet R.P., Layé S. (2014). Polyunsaturated fatty acids and their metabolites in brain function and disease. Nat. Rev. Neurosci..

[43] Ren H., Luo C., Feng Y., Yao X., Shi Z., Liang F., Kang J.X., Wan J.B., Pei Z., Su H. (2017). Omega-3 polyunsaturated fatty acids promote amyloid-beta clearance from the brain through mediating the function of the glymphatic system. FASEB J. Off. Publ. Fed. Am. Soc. Exp. Biol..

[44] Cheng P.-F., Chen J.-J., Zhou X.-Y., Ren Y.-F., Huang W., Zhou J.-J., Xie P. (2015). Do soy isoflavones improve cognitive function in postmenopausal women? A meta-analysis. Menopause.

[45] Cui C., Birru R., Snitz B.E., Ihara M., Lopresti B.J., Aizenstein H.J., Lopez O.L., Mathis C., Miyamoto Y., Kuller L.H. (2018). Effects of Soy Isoflavones on Cognitive Function: A Systematic Review and Meta-Analysis of Randomized Controlled Trials. Alzheimers Dement..

[46] Man B., Cui C., Zhang X., Sugiyama D., Barinas-Mitchell E., Sekikawa A. (2020). The effect of soy isoflavones on arterial stiffness: A systematic review and meta-analysis of randomized controlled trials. Eur. J. Nutr..

[47] Hughes T.M., Kuller L.H., Barinas-Mitchell E.J., Mackey R.H., McDade E.M., Klunk W.E., Aizenstein H.J., Cohen A.D., Snitz B.E., Mathis C.A. (2013). Pulse wave velocity is associated with beta-amyloid deposition in the brains of very elderly adults. Neurology.

[48] Cui C., Sekikawa A., Kuller L.H., Lopez O.L., Newman A.B., Kuipers A.L., Mackey R.H. (2018). Aortic Stiffness is Associated with Increased Risk of Incident Dementia in Older Adults. J. Alzheimers Dis..

[49] Strazzullo P., D’Elia L., Kandala N.-B., Cappuccio F.P. (2009). Salt intake, stroke, and cardiovascular disease: Meta-analysis of prospective studies. BMJ.

[50] Yamazaki Y., Zhao N., Caulfield T.R., Liu C.-C., Bu G. (2019). Apolipoprotein E and Alzheimer disease: Pathobiology and targeting strategies. Nat. Rev. Neurol..

[51] Villain N., Chetelat G., Grassiot B., Bourgeat P., Jones G., Ellis K.A., Ames D., Martins R.N., Eustache F., Salvado O. (2012). Regional dynamics of amyloid-beta deposition in healthy elderly, mild cognitive impairment and Alzheimer’s disease: A voxelwise PiB-PET longitudinal study. Brain J. Neurol..

[52] Kim E.S., Konrath S. (2016). Volunteering is prospectively associated with health care use among older adults. Soc. Sci. Med..

[53] Roberts R.O., Aakre J.A., Kremers W.K., Vassilaki M., Knopman D.S., Mielke M., Alhurani R., Geda Y.E., Machulda M.M., Coloma P. (2018). Prevalence and Outcomes of Amyloid Positivity among Persons without Dementia in a Longitudinal, Population-Based Setting. JAMA Neurol..

